# Sulfotransferase 4A1 activity facilitates sulfate-dependent cellular protection to oxidative stress

**DOI:** 10.1038/s41598-022-05582-4

**Published:** 2022-01-31

**Authors:** Evan J. Brettrager, Arthur W. Meehan, Charles N. Falany, Robert C. A. M. van Waardenburg

**Affiliations:** Department of Pharmacology and Toxicology, University of Alabama at Birmingham, 155 Volker Hall, 1720 2nd Ave S., Birmingham, AL 35294-0019 USA

**Keywords:** Cell biology, Neuroscience

## Abstract

Sulfotransferase 4A1 (SULT4A1) is an orphan member of the cytosolic SULT superfamily that contains enzymes that catalyze the sulfonation of hydrophobic drugs and hormones. SULT4A1 has been assessed through all classical SULT approaches yet no SULT activity has been reported. To ascertain SULT4A1 function and activity, we utilized *Saccharomyces cerevisiae* as a model system, which exhibits no endogenous SULT activity nor possesses SULT-related genes. We observed that ectopic SULT4A1 expression in yeast displays similar subcellular localization as reported in mouse neurons and observed that SULT4A1 is associated with the outer mitochondria membrane. SULT4A1 expression stimulates colony formation and protects these cells from hydrogen peroxide and metabolism-associated oxidative stress. These SULT4A1-mediated phenotypes are dependent on extracellular sulfate that is converted in yeast to PAPS, the universal sulfonate donor for SULT activity. Thus, heterologous SULT4A1 expression in yeast is correctly distributed and functional, and SULT4A1 antioxidant activity is sulfate dependent supporting the concept that SULT4A1 has sulfate-associated activity.

## Introduction

Sulfotransferase 4A1 (SULT4A1) was initially identified and cloned from human and rat brain two decades ago^1^. SULTs are considered Phase 2 drug metabolizing enzymes that catalyze the sulfonation of hydrophobic drugs and hormones, which transforms these molecules into hydrophilic metabolites, to regulate their cellular activity and excretion^2^. SULT4A1 is a member of the cytosolic SULT superfamily based on sequence and protein structural homology with other SULTs^1,3^. The SULT4A1 amino acid sequence is highly conserved within vertebrates^4^ and no homologous SULT4A1 sequences have been reported in invertebrates. Moreover, the human SULT4A1 gene shows an unusually low rate of mutation and exhibits the lowest mutation frequency among all known human SULTs^5,6^. Most SULT isoforms are widely expressed in tissues; however, SULT4A1 protein is primarily detected in neurons of the central nervous system (CNS) of humans, rats, and mice^1,2,4,7–12^. The SULT4A1 CNS expression pattern and growing reports strongly suggest a critical function for SULT4A1 in neuronal development and function. Disruption of SULT4A1 expression contributes to neurodevelopmental syndromes; Sequence polymorphisms in the 5’-UTR and haploinsufficiency of the SULT4A1 gene (deletions at loci 22q13.3) are associated with schizophrenia and Phelan-McDermid syndrome, an autism spectrum disorder^13–17^. The first direct SULT4A1 phenotypes came from studies utilizing zebrafish, revealing possible roles in phototransduction and excessive sedentary behavior during day(light)-time^4,18^. Subsequent generation of mouse *sult4A1*-knockout (KO) models showed a severe neurological phenotype that resulted in death 3 to 4 weeks after birth^7^. Although no activity has been described for SULT4A1, the protein in fact has an essential role in normal neuronal development^7,9,19^. SULT4A1, unlike other cytosolic SULTs, localizes to the cytosolic and mitochondrial subcellular fractions of mouse and human brains^7^. This suggests that SULT4A1 might have a supportive role in mitochondrial function. Indeed, Hossain et al. reported that ectopic expressed SULT4A1 has a direct regulatory role in mitochondria function and redox-homeostasis^9^. These observations could explain the critical regulatory role of SULT4A1 in neuronal cell populations that exhibit a remarkably high-energy demand and generate increased levels of oxidative stress in the form of reactive oxygen species. Due to the specific neuronal expression of SULT4A1, characterizing its activity in primary neurons and cultured neurons presents certain obstacles. Most importantly these models express additional SULTs that hinder identification of specific SULT4A1 sulfonation substrates^20^. We use the tractable eukaryotic single cell model organism *Saccharomyces cerevisiae* in our quest to ascertain SULT4A1 activity and function. Yeast is an established model organism to investigate function, activity, and post-translational modification of neuronal proteins and mitochondrial disease mechanisms^21–23^. Moreover, the yeast genome does not contain any homologous SULT sequences and yeast cells do not show any sulfonation activity^24^. Thus, SULT4A1 expression in yeast provides a clean model system including endogenously produced 3'-phosphoadenosine-5'-phosphosulfate (PAPS) from imported environmental sulfate for use in Met and Cys synthesis^24^. Herein, we report that ectopic SULT4A1 expression in yeast displays a similar subcellular cytosolic and mitochondrial distribution as mouse neurons and cultured neuronal cell models^7,9^. Moreover, we show that SULT4A1 is associated with the mitochondrial outer membrane. In addition, SULT4A1 expression protects yeast cells from hydrogen peroxide induced toxicity and metabolically generated oxidative stress. Strikingly, the SULT4A1 mediated protective and growth stimulating phenotypes are sulfate dependent, suggesting that SULT4A1 exhibits functional sulfate-activity.

## Results

### Ectopic expression of SULT4A1 in yeast forms a stable functional protein that stimulates cell growth

**Figure 1:**
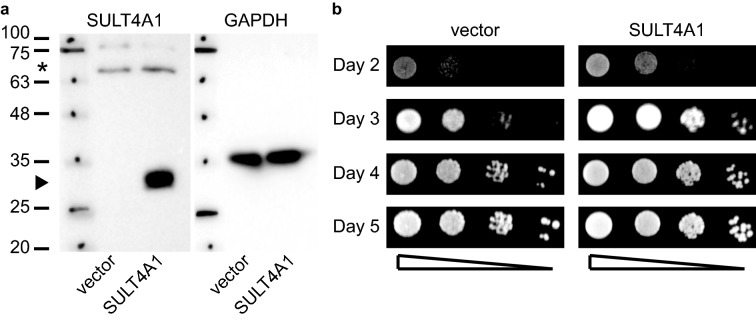
SULT4A1 expression in yeast stimulates colony formation. Galactose induced exponentially growing yeast cells transformed with YCpGAL1*SULT4A1*•L (SULT4A1) or control (vector) were used for (**a**) isolation of total cell extracts. 20 μg of protein of each were resolved via SDS-PAGE and transferred to a PVDF membrane, immunostained for SULT4A1 followed by stripping and immunostaining for loading control proteins; GAPDH-cytosol. Arrowhead points at SULT4A1, *non-specific staining of anti-SULT4A1 antibody. Protein ladder with sizes in kDa. (**b**) Cell cultures from yeast transformants (**a**) were corrected to OD_595_ = 0.3, ten-fold serially diluted and spotted onto 2% galactose selective media plates and incubated at 30 °C. Growth was recorded from day 2 to 5. Shown is a representative result from at least 3 independent experiments.

SULT4A1 is highly conserved and is selectively expressed in neuronal tissue in vertebrates with a potential role in supporting mitochondrial function and protects against oxidative stress/toxicity^1,3,7–11^. Yet, no SULT4A1 catalytic activity has been detected using many classical SULT approaches^1,3,4,25^. To ascertain the activity and function of SULT4A1, we utilized the yeast *S. cerevisiae* as a genetically tractable single cell organism that is a well-recognized model organism to study mitochondria and neuronal protein functions. Moreover, studying SULT4A1 in yeast has the advantage that yeast cells innately produce 3'-phosphoadenosine-5'-phosphosulfate (PAPS), solely for the biosynthesis of Met and Cys residues^24^. Since yeast does not exhibit SULT activity, we first needed to demonstrate that yeast can tolerate heterologous SULT4A1 expression and its potential cellular activity. To prevent potential adverse effects of ectopic SULT4A1, we used plasmid-borne galactose-inducible SULT4A1 expression. The *GAL1* promotor is actively repressed when yeast is cultured in dextrose and becomes transcriptionally active when yeast is cultured in the presence of galactose. Other carbon sources are considered ‘neutral’ as they do not repress or induce transcription from the *GAL1* promotor. Yeast cultures with and without SULT4A1 did not show any adverse effects when cultured in dextrose media (no expression) nor in galactose media (induced expression). Subsequent analysis of total cell extracts of galactose induced cultures of cells with and without SULT4A1 revealed that SULT4A1 forms a stable full-length protein (Fig. 1a). This suggests that yeast can tolerate galactose induced SULT4A1 expression and potential SULT4A1-mediated activity. Indeed, cell viability experiments showed that expression of SULT4A1 stimulates colony formation compared to vector control (no SULT4A1) over a 5-day time span (Fig. 1b). These observations indicate that yeast expresses SULT4A1 at robust levels without any SULT4A1-related cytotoxic effects. Instead, SULT4A1 expression stimulates yeast colony formation suggesting that SULT4A1 is properly folded and has growth stimulatory functions following expression in yeast.

### SULT4A1 subcellular distribution in yeast is similar to mouse neuronal cells

**Figure 2:**
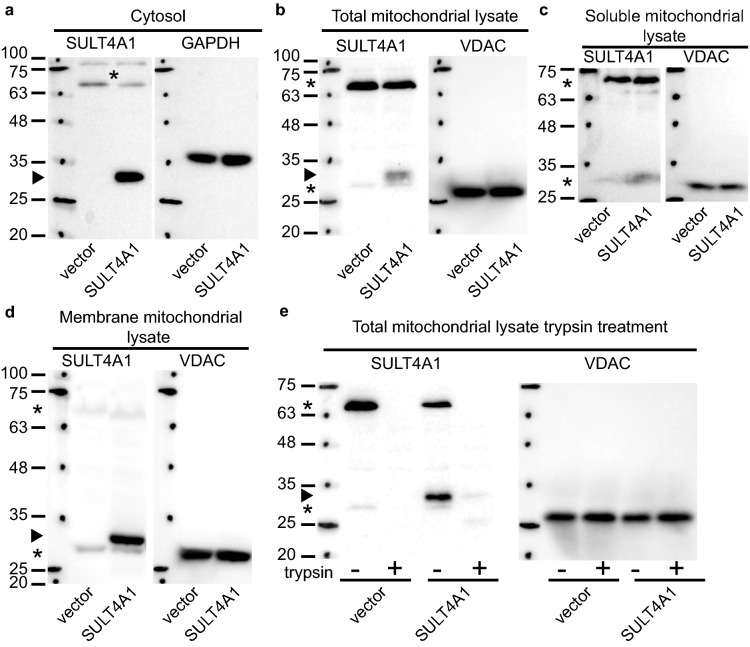
SULT4A1 subcellular distribution in yeast. Yeast transformed with YCpGAL1*SULT4A1*•L (SULT4A1) or control (vector) were exponentially grown in galactose for 36 h. Spheroplasts were generated and subcellular fractions isolated via differential centrifugation and sucrose gradient resolution of spheroplasts lysates. 20 μg of protein of each were resolved via SDS-PAGE and transferred to a PVDF membrane followed by immunostaining for SULT4A1 followed by stripping and immunostaining for loading control proteins; GAPDH-cytosol; VDAC1-mitochondria. (**a**) cytosol; (**b**) total mitochondrial lysate; (**c**) purified mitochondrial soluble lysate and (**d**) insoluble mitochondrial membrane fraction; (**e**) total lysates of purified mitochondrial fraction with ( +) or without (-) trypsin treatment. Arrowhead points at SULT4A1, *: non-specific staining of anti-SULT4A1 antibody. Protein ladder with sizes in kDa.

To further validate yeast as an appropriate model to study SULT4A1 activity and function, we determined the subcellular distribution of SULT4A1 protein in yeast. In neuronal cells SULT4A1 is located in the cytosol, like all cytosolic SULTs^7^. However, unlike the other SULTs, SULT4A1 was also located in the mitochondria and microsomal (membrane vesicle-like artifacts formed during tissue/cell homogenization) fractions. Exponentially growing cells, with or without SULT4A1, were induced with galactose for 36 h to induce robust SULT4A1 expression. We subsequently isolated the cytosolic and mitochondrial fractions from yeast spheroplast lysates via differential centrifugation and separation using a sucrose gradient. SULT4A1 was detected in cytosol and a weak signal was observed in the lysate of the mitochondrial fraction (Fig. 2a and b). Next, we ascertained the location of SULT4A1 in purified mitochondria. SULT4A1 is associated with the mitochondrial membrane fraction and not present in the soluble (matrix or inner membrane space) fraction (Fig. 2c and d). Overall, these results indicate that SULT4A1 migrates to the same subcellular locations in yeast as reported in neuronal cells^7,9^.

### SULT4A1 is associated with the mitochondrial outer membrane on the cytosolic side

Although, we detected that SULT4A1 is associated with the membrane fraction, this observation does not accurately define to which mitochondrial membrane (outer or inner) SULT4A1 is associated and on which side of these membranes (cytosolic, inner space or matrix). To examine SULT4A1 association with the mitochondria outer membrane, we treated purified mitochondria with trypsin to proteolyze outer mitochondrial proteins and peptides before isolating total mitochondria lysates. Trypsin treatment degraded all SULT4A1 but not the VADC/porin1 loading control and SULT4A1 was detected in the control sample—no trypsin (Fig. 2e). These results suggest that SULT4A1 is uniquely associated with the mitochondrial outer membrane at the cytosolic site and is not imbedded in the mitochondrial outer membrane as is VDAC/porin1^26^ or associated with the mitochondrial inner membrane.

### SULT4A1 expression protects yeast cells from hydrogen peroxide-induced oxidative stress

**Figure 3:**
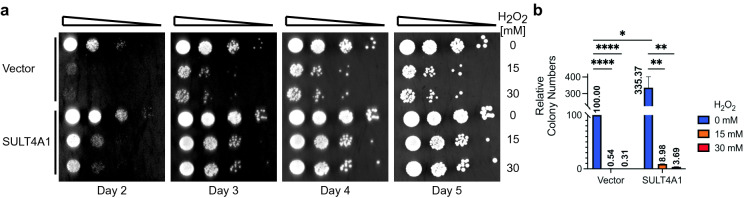
SULT4A1 protects yeast from H_2_O_2_ induced cytotoxicity. Exponentially growing galactose-induced yeast transformed with YCpGAL1*SULT4A1*•L (SULT4A1) or control (vector) were aliquoted and treated with 0, 15 and 30 mM H_2_O_2_ for 1 h at 30 °C, cultures were corrected to OD_595_ of 0.3 and tenfold serial diluted. (**a**) Dilutions were spotted on galactose plates, incubated at 30 °C, and depicted is a representative picture from day 2 to 5. (**b**) Appropriate dilutions were spread on galactose plates and number of colonies were determined after 4 days at 30 °C. Depicted is the mean and SEM of relative colony number to vector control with no H_2_O_2_ treatment of three independent experiments (relative colony number is shown in graph). Unpaired (two-tailed) t-test by Prism; *P < 0.05, **P < 0.01, ****P < 0.0001.

Recently the first functional phenotypes of ectopic SULT4A1 expression were reported; SULT4A1 transduced human SH-SY5Y neuroblastoma cells showed reduced sensitivity to H_2_O_2_-induced oxidative stress^9^. For yeast to be a functional model organism to decipher SULT4A1 activity and function, SULT4A1 should protect yeast cells from H_2_O_2_ induced oxidative toxicity. We first determined yeast sensitivity to H_2_O_2_ grown on galactose and dextrose. Independent of the carbon source, yeast cells start to show H_2_O_2_ sensitivity at concentrations > 10 mM under our growth conditions. Interestingly, the protective function of SULT4A1 expression in yeast is effective and ostensibly independent of H_2_O_2_ concentration (Fig. 3). The no treatment controls corroborated the growth stimulative effect of SULT4A1 expression shown in spot tests (Fig. 1a). Quantitive colony formation shows that cells expressing SULT4A1 grow 3 times the number of colonies as cells without SULT4A1 expression (Fig. 3b). Moreover, SULT4A1 expressing cells are on average ~ 15 times less sensitive to H_2_O_2_ induced oxidative stress than cells without SULT4A1 (Fig. 3b). These results suggest that ectopic expression of SULT4A1 in yeast protects cells from H_2_O_2_ induced cytotoxicity as compared to cells without SULT4A1 expression.

### SULT4A1 protects yeast from metabolically generated oxidative stress

**Figure 4:**
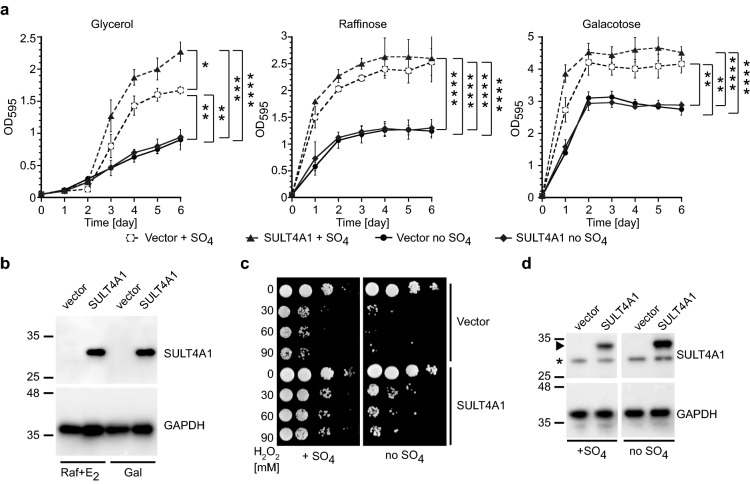
SULT4A1 protection to metabolically generated and H_2_O_2_ induced oxidative toxicity is sulfate dependent. Yeast transformed with YCpGAL1*SULT4A1*•L (triangle and diamond) or vector control (open and closed circle) were cultured in media supplemented with (**a**) glycerol with 1 μg/ml E2; raffinose with 1 μg/ml E2 and galactose, with (dotted line) or without (solid line) additional ammonium sulfate (SO_4_). Exponentially growing cultures were diluted to an OD_595_ of 0.05 at day 0 and incubated at 30 °C. OD_595_ was determined every day for 6 days. Shown is the mean and SD of three independent experiments. One way ANOVA followed by Tukey multiple comparison test using Prism; *P < 0.05, **P < 0.01, ***P < 0.001, ****P < 0.0001. (**b**) Yeast transformed with YCpGAL1*SULT4A1*•L (SULT4A1) or control (vector) were induced for 36 h in galactose or raffinose + 1 μg/ml E2 and total lysates were isolated from exponentially growing cultures. 20 μg of protein of each was resolved via SDS-PAGE and transferred to a PVDF membrane followed by immunostaining for SULT4A1 followed by stripping and immunostaining for the loading control GAPDH. Protein ladder with sizes in kDa. (**c**) Galactose-induced exponentially growing cultures, with or without additional ammonium sulfate, of yeast transformed with YCpGAL1*SULT4A1*•L (SULT4A1) or control (vector) were aliquoted and treated with 0, 30, 60 and 90 mM H_2_O_2_ for 1 h at 30 °C, corrected to an OD_595_ of 0.3, tenfold serial diluted and spotted on galactose plates with or without additional ammonium sulfate, incubated at 30 °C. (**d**) Yeast transformed with YCpGAL1*SULT4A1*•L (SULT4A1) or control (vector) were induced for 36 h in galactose with or without additional sulfate-salt and total lysates were isolated from exponentially growing cultures. 20 μg of protein of each were resolved via SDS-PAGE and transferred to a PVDF membrane followed by immunostaining for SULT4A1 followed by stripping and immunostaining for the loading control GAPDH. Arrowhead points at SULT4A1, *non-specific staining of anti-SULT4A1 antibody. Protein ladder with sizes in KDa.

SULT4A1 expression protects yeast cells against exogenous H_2_O_2_- induced oxidative stress. This prompts the question; If one of SULT4A1 physiological roles is to protect cells from metabolically generated oxidative damage? In humans, SULT4A1 protein is predominately found in neurons, which produce a high level of metabolically generated oxidative stress to fulfill their energy (ATP) requirements via respiration/oxidative phosphorylation^1,7,9,27^. Yeast cells prefer to use the energetically less efficient fermentation process over the energy efficient oxidative phosphorylation pathways^28^. Yeast cells using fermentation have lower levels of metabolically produced ROS^28,29^. Lower ROS-levels reduces the amount of oxidative damage to lipids, proteins, and nucleic acids resulting in prolonged proliferation and potentially increased cell lifespan^28,29^. We examined SULT4A1’s role in protecting cells against metabolically generated ROS by monitoring growth in liquid media supplemented with glycerol as non-fermentable carbon source, and the fermentable carbon sources, raffinose and galactose. Glycerol and raffinose, unlike galactose or dextrose, do not activate or repress transcription of the *GAL1* promoter. We induced expression of SULT4A1 by co-expressing the chimeric Gal4-ER-VP16 transcription activator^30^. This chimeric transcription factor contains the Gal4 DNA binding domain that binds to its ‘upstream activating sequence’ in the *GAL1* promoter, which forms an active transcription factor upon exposure to estradiol. Estradiol stimulates dimerization via the estrogen receptor dimerization domain (ER) while the VP16 transcription activation domain stimulates transcription. Thus, in the presence of estradiol this chimeric transcription factor stimulates transcription from the *GAL1* promoter^30^. Yeast expressing SULT4A1 grow significantly better than cells without SULT4A1 with glycerol as a carbon source (Fig. 4a-dotted lines). Also, media supplemented with raffinose or galactose display a less pronounced SULT4A1 growth stimulation in liquid media (Fig. 4a-dotted lines) compared to colony formation assay (Figs. 1b and 3). The SULT4A1 protein levels between galactose and raffinose/estradiol-*Gal4-ER-VP16* stimulated expression do not display gross differences at their relative expression levels (Fig. 4b). Although galactose and raffinose are (poor) fermentable sugars, their metabolism increases ROS levels compared to the fermentation of dextrose^28,29^. Overall, these observations suggest that SULT4A1 protects cells from metabolically generated oxidative stress and cell death.

### SULT4A1 protection from hydrogen peroxide and metabolically generated oxidative damage is sulfate dependent

Since no SULT activity for SULT4A1 has been reported, we studied the effect of omitting sulfate from the media on the SULT4A1 phenotypes described above. Omitting sulfate from the media prevents yeast from producing PAPS^24^, needed for SULT-mediated activity^2,31^. We first examined the sulfate-dependency of SULT4A1 stimulated cell growth and protective function to metabolically generated oxidative damage shown in Fig. 4a glycerol-dotted lines. Eliminating sulfate impeded SULT4A1 from providing growth stimulation or protection to metabolically generated oxidative damage (Fig. 4a-solid lines). Next, we assessed the sulfate-dependency of SULT4A1 mediated H_2_O_2_ resistance. Yeast cells with and without SULT4A1 expression were treated with different concentrations of H_2_O_2_ in media with and without added sulfate (Fig. 4c). We increased the range of H_2_O_2_ from 15–30 mM to 30–90 mM to examine a more dynamic range. SULT4A1 expression with sulfate protects yeast from 90 mM H_2_O_2_ induced toxicity displayed by the control cells (Fig. 4c). Conversely, SULT4A1 expressing cells grown without additional sulfate showed an increased sensitivity compared to cells with sulfate but are not as sensitive as cells without SUL4A1 (Fig. 4c, d). The residual H_2_O_2_ ‘resistance’ by SULT4A1 is probably due to the low level of sulfate-salts (~ 0.5 g/L) present in the YNB mix. Omitting sulfate-salt from the media did not affect SULT4A1 expression levels indicating that these phenotypes are SULT4A1-dependent. These results suggest that the protective function of SULT4A1 to endogenous and exogenous generated oxidative damage is dependent on sulfate and by extension PAPS.

## Discussion

SULT4A1 was identified and isolated 20 years ago^1^ and is still an intriguing, unique isoform within the cytosolic SULT family. Although, SULT4A1 contains the highly conserved SULT-catalytic Lys-Lys-His residues, no sulfation activity by SULT4A1 for small (physiological) compounds has been reported^1,3,4,25^. A recent report suggested that SULT4A1 is able to sulfonate 1-napthol using the *S. pombe* ‘enzyme’ bag assay, yet this observation has not been reproduced by other laboratories^32^. Fission yeast and budding yeast do not possess any endogenous SULT-activity. SULT4A1 does appear to have multiple important functions in the central nervous system. In family studies, SULT4A1 shows transmission disequilibrium with the occurrence of schizophrenia^16,17,33^. SULT4A1 gene deletion has also been linked to Phelan-McDermid Syndrome (PMS), a generalized cognitive and developmental autism spectrum syndrome^13,14^. The first direct SULT4A1 phenotypes came from studies utilizing zebrafish that showed a role for SULT4A1 in expression of cone genes in phototransduction^4^, and regulation of activity levels during daylight^18^. Subsequent generation of homozygous Δ12 and Δ28 knockout mice, showed SULT4A1 selective expression to neurons with a cytosol and mitochondrial subcellular localization that is unique for cytosolic SULTs^7,8,12^. Moreover, SULT4A1 knockout pups of both sexes develop significant tremors, movement/balance issues, dysmorphic changes in snout and back, failure to thrive, bouts of apparent silent seizures, become immobile, and die or are euthanized between 21 and 24 days of age^7^. Subsequent studies showed that SULT4A1 protects cells against oxidative stress induced toxicity, supports mitochondria function and redox-homeostasis in mice cultured cortical neurons and SULT4A1 transduced SH-SY5Y cells^9^. Nevertheless, the catalytic activity of SULT4A1 remains an enigma^1,3,4,25^.

SULT4A1 is highly conserved among vertebrates and no homologous gene sequences are reported in invertebrates. Moreover, SULT4A1 shows the lowest number of SNPs in humans of all cytosolic SULTs^5,6^. These observations suggest that SULT4A1 function and activity is highly conserved and includes potential interfaces required for SULT4A1-protein interactions. As such we reasoned that baker’s yeast might be a good model organism to ascertain SULT4A1 function and activity. Yeast does not contain any homologous SULT gene sequences and does not show any native SULT activity^24^. Yeast does natively produce PAPS (for Met and Cys biosynthesis), which is the sulfonate donor for all cytosolic SULTs^1,2,24^. Moreover, yeast is a tested model organism to study neuronal protein function and activity^21–23^. As such, we verified that expression of heterologous SULT4A1 produces a stable protein and does not induce toxicity in yeast. Moreover, SULT4A1 subcellular distribution is similar to that reported in mouse brain and neuronal cells^7,9^. We observed that SULT4A1 is localized in the subcellular cytosolic and mitochondrial fractions of yeast lysates and the artificially formed microsomal fraction (data not shown). These observations suggest that the ectopic expressed SULT4A1 in yeast is properly folded, localized to similar subcellular locations, and is functional. Further dissection of SULT4A1 association with the mitochondrial fraction showed SULT4A1 is associated with the mitochondrial membrane fraction and not with its soluble fractions. Membrane localization was suggested by Hossain et al. in their orthogonal projection of fluorescent immunostaining that showed partial colocalization with TOM70 in SULT4A1 transduced SH-SY5Y cells and intense mitochondrial localization of ectopic SULT4A1^9^. To dissect SULT4A1 mitochondrial membrane localization, we treated the sucrose gradient purified mitochondrial fraction with trypsin. We observed that SULT4A1 is associated with the mitochondrial outer membrane and not located within the membrane like VDAC/Porin1 as this protein was not affected by trypsin treatment. This allows a more focused approach to ascertain how SULT4A1 is associated with the outer membrane and how it stimulates mitochondrial function, which seems to be a conserved event from yeast to vertebrate neurons.

Although, ectopic SULT4A1 is stably expressed and exhibits a similar subcellular localization as in neuronal cells, we needed to verify that this heterologous SULT4A1 protein functions in yeast as in neuronal cells. One testable function reported thus far is SULT4A1 protection from oxidative-H_2_O_2_-induced stress/toxicity^9^. Ectopic expression of SULT4A1 protects yeast cells from H_2_O_2_ induced toxicity. In addition, SULT4A1 expression stimulates yeast colony formation and growth in liquid media under fermentative (mitochondrial independent generation of ATP) and respiratory (mitochondria dependent generation of ATP) growth conditions. This SULT4A1 stimulated growth advantage in yeast could be related to similar events recently report by Culotta et al.^34^. These authors reported that SULT4A1 is involved in regulating neuronal branching and dendritic spine formation, which was significantly reduced in cells that were SULT4A1 deprived^34^. SULT4A1 provides a significant growth advantage under respiratory growth conditions that is potentially related to protection against increased levels of mitochondria generated reactive oxygen species (ROS) including H_2_O_2_ production. Yet, even under low mitochondrial produced ROS (fermentation) SULT4A1 granted a growth advantage to cells. Yeast fermentation reactions are similar to the Warburg effect known to provide advantageous growth conditions for cancer cells under low oxygen conditions^35^. However, this Warburg effect or aerobic glycolysis has also been reported to exists in specific areas of the brain where the glucose utilization exceeds the oxygen consumption and is associated with Amyloid beta resistance^36–38^. These yeast observations could be an indication that SULT4A1 might protect mitochondria and stimulate neuronal cell propagation during aerobic glycolysis that peaks during early childhood and becomes more restricted to specific areas during adulthood^37,38^. The question remains, how does SULT4A1 mitigate the effects of ROS and peroxide? Part of the answer is that SULT4A1 protective and growth stimulating effect is dependent on sulfate. We omitted the majority sulfate from the media under fermentative and respiratory growth conditions that diminished the growth advantage and protection to H_2_O_2_ induced stress/toxicity provided by SULT4A1 expression. Omitting sulfate from the media did not affect SULT4A1 protein levels, signifying that the observed phenotypes are SULT4A1 activity dependent. The residual protection of SULT4A1 to H_2_O_2_ is due to the low levels (~ 0.5 g/L) of sulfate present in the commercial yeast nitrogen base without amino acids without ammonium sulfate mix (BD Difco) in the form of copper-, manganese-, zinc- and magnesium-sulfate salts. These results suggest that SULT4A1 possesses SULT activity. In yeast, sulfate is converted into the universal SULT sulfonate donor PAPS that in yeast is solely used as the sulfur donor for the synthesis of Met and Cys.

In Summary, the observations herein show that heterologous expression of SULT4A1 in yeast stimulates yeast growth under fermentative (aerobic glycolysis or Warburg effect) and respiratory growth conditions and protects cells from metabolically generated and exogenously induced oxidative stress/toxicity. All these SULT4A1 mediated phenotypes are sulfate-dependent, implying that they are dependent on SULT4A1 SULT activity. Additionally, ectopic SULT4A1 displays a similar subcellular distribution to the cytosol and the mitochondria as reported in neuronal cells, and that SULT4A1 is associated with the outer mitochondrial membrane. These observations suggest that SULT4A1 expression in yeast supports mitochondria function and regulates redox-homeostasis and protects against oxidative stress induced toxicity that are all dependent on sulfate. This implies that the interaction interfaces SULT4A1 used to associate with the mitochondrial outer membrane and cytosolic interaction partners are highly conserved from vertebrates to yeast. However, the question remains, how does SULT4A1 facilitate these sulfate-dependent phenotypes? Independent of what SULT4A1 activity is, its potential substrates and/or interaction partners exist in yeast and seemed to be conserved in vertebrate neurons. Thus, our observations reported here suggests that *Saccharomyces cerevisiae* is an appropriate model organism to investigate SULT4A1 function, activity, and its molecular mechanism of action.

## Materials and methods

### Yeast strain and plasmid

*Saccharomyces cerevisiae* strain MGY-250 (*MATα, ura3∆::LoxP, his3A200, leu2Al, trp1∆63*) was generated from FY-250 (*MATα, ura3-52, his3A200, leu2Al, trp1∆63*) by gene replacement of the *ura3-52* allele with LoxP-*KAN*^r^-LoxP, followed by CRE-mediated recombination to yield *ura3∆::LoxP*^39^. The murine *SULT4A1* open reading frame sequence was PCR amplified from PLVXmSULT4A1-Puro plasmid (generous gift from Dr. Andrabi^9^) with *Bam*HI-*Xba*I ends (forward primer; 5′-GCCGGATCCATGGCGGAGAGCG AAGCG-3′ and reverse primer 5′-GGCTCTAGATTATAGATAAAAGTCAAACGTGAGGTC-3′) and cloned via directed gene replacement of *TDP1* into YCpGAL1*TDP1*•L (*LEU2*) vector resulting in pRS415GAL1mSULT4A1•LEU2 (YCpGAL1*SULT4A1*•L)^39^. Plasmid born mouse SULT4A1 was expressed from the galactose inducible (*GAL1*) promotor to prevent adaptation or cytotoxic effects of the heterologous SULT4A1 protein. To express SULT4A1 from the *GAL1* promoter under growth conditions using non-inducible carbon sources (raffinose or glycerol), we co-expressed the chimeric transcription-activator GAL4-ER-VP16 from the pRS313*ADH1*HA-GAL4ERVP16-Flag•HIS3 plasmid (generous gift from Dr. Kodadek^30^) and induce dimerization of the chimeric transcription factor with 1 μg/ml estradiol (E_2_) to obtain transactivation. All minimal media contains yeast nitrogen base without amino acids and ammonium sulfate (BD Difco), supplemented with essential amino acids mix without those used for autotroph selection, indicated carbon source, and with 5 g/L ammonium sulfate except when specially noted without sulfate. In all cases, gene deletions were confirmed by PCR followed by DNA sequencing and cloned alleles were verified by DNA sequencing. All experiments were independently repeated at least three times, and a representative experiment is depicted in “Results” section.

### Yeast cell viability assays

Cultures of yeast cells transformed with the indicated vectors were grown overnight at 30 °C in selective minimal media supplemented with 2% dextrose, diluted 1:100 in selective minimal media supplemented with 2% galactose, or with 2% raffinose or 3% glycerol and grown overnight at 30 °C. SLUT4A1 expression is induced with 1 μg/ml E2 in cultures supplemented with raffinose and glycerol. These overnight cultures were subsequently used for the following cell viability assays:

(1) Spot test or colony formation assay. Overnight cultures were diluted to OD_595_ of 0.25 in minimal selective media with the selected carbon source and grown until OD_595_ ~ 0.6 to obtain exponentially growing cultures. For H_2_O_2_ toxicity spot test, these exponentially growing cultures were aliquoted and incubated for 1 h with the indicated concentration of H_2_O_2_ at 30 °C. Exponentially growing (treated) cultures were diluted to OD_595_ of 0.3 in TE buffer [50 mM Tris (pH 8.0), 5 mM EDTA] and tenfold serially diluted, and 5 µl aliquots were spotted onto selective media plates containing indicated carbon source. Plates were incubated for 7 days at 30 °C with growth being recoded every day using a gel-doc system (SYNGENE G:Box). All images were processed via Adobe Photoshop 2021 to correct signal levels of the complete image before cropping the shown area and placed into Adobe Illustrator 2021 to generate final figures.

(2) Quantitative colony formation assay. To quantify H_2_O_2_ toxicity, two selective media plates with indicated carbon sources were each spread with 50 µl of the appropriate dilution of treated cultures (as described in *Spot test*) and incubated for 4 days at 30 °C and colonies were counted by hand. At least three independent assays were used, graphed, and analyzed using unpaired (two-tailed) t-test using Prism.

(3) Liquid growth curves. The overnight cultures were diluted to an OD_595_ of 0.05 in 5 ml selective media with the appropriate carbon source with estradiol when needed and grown at 30 °C. Every 24 h for 6 days the culture’s OD_595_ were determined. Results of at least three independent assays were used, graphed and one way ANOVA followed by Tukey multiple comparison test of cell starting exponential growth phase using Prism.

### Isolation of total cell extracts

Yeast cells transformed with the indicated vectors were grown as described in the *yeast cell viability assay* with the indicated carbon source and harvested on day 3 at an OD_595_ of 0.6–0.8, washed in cold sterile deionized water, and cell pellet was resuspended in 150 µL TEEG buffer [50 mM Tris pH 8.0, 2 mM EDTA, 2 mM EGTA, 10% glycerol] with 1% sodium deoxycholate, 1 mM phenylmethylsulfonyl fluoride (PMSF), protease inhibitor-EDTA free (Pierce), and 100 µL frozen (− 20 °C) sterilized acid-washed glass beads. The samples were lysed at 4 °C in a bead beater, 10 cycles of 30 s on, 1 min off. The lysate was cleared from cell debris/glass beads and the supernatant fractions were extracted, and protein concentrations determined by Bradford assay. Lysate was boiled in SDS buffer for 10 min and stored at − 20 °C or immediately used for immunoblotting/staining.

### Isolation of subcellular fractionations

Yeast cells transformed with the indicated vectors were grown as described in the *yeast cell viability assay* and exponentially growing galactose induced cells were harvested and resuspended in Zymolyase Buffer (50 mM Tris–HCl pH 7.5, 10 mM MgCl_2_, 25 mM EDTA, 1 M Sorbitol, 30 mM DTT, 1 mM PMSF), flash frozen in liquid nitrogen and stored at − 80 °C. Thawed cells were dosed with additional 1 mM PMSF and 200 µg/ml Zymolyase T-20 (Nacalai Tesque, inc), incubated at 36 °C with gentle agitation for 1.5 h to generate spheroplasts. All following manipulations were done on ice or at 4 °C. Spheroplasts were harvested and resuspended in Subcell Frac Buffer (20 mM HEPES pH8, 10 mM NaCl, 1 mM EDTA, 1 mM EGTA, 1 mM DTT, 1 mM PMSF) and lysed with 20 strokes in a glass Dounce homogenizer. The lysate was cleared of whole cells and large debris by repeated 200 RCF centrifugations and the crude mitochondria was pelleted from this cleared lysate. The supernate of this fraction was cleared by ultracentrifugation at 134,000 RCF for 1 h to generate the cytosolic fraction and microsomal (pellet) fraction. The crude mitochondria pellet was resuspended in 1 mL Subcell Frac Buffer and subjected to ultracentrifugation over a sucrose gradient (2 mL 32% sucrose layered over 1.5 mL 70% sucrose) at 134,000 RCF for 1 h. Pure mitochondria were recovered from the S70-band that formed between the 32 and 70% sucrose layers of the gradient. The pure mitochondria were washed in Subcell Frac Buffer then resuspended in Lysis Buffer (10 mM HEPES pH8, 550 mM NaCl, 0.1 mM EDTA, 0.1 mM EGTA, 1% TritonX-100, 1% Sodium deoxycholate, 1 mM DTT, 1 mM PMSF). Mitochondria were lysed by vortexing hard 15 times for 15 s then sonicated 5 times 5 s at 20% output. Mitochondrial lysates were cleared by centrifugation at 17,000 RCF for 30 min and the supernatant fractions were collected. Bradford assays were conducted on all samples to determine protein concentrations and lysates were boiled in SDS buffer for 10 min and were stored at − 20 °C or immediately used for immunoblotting/staining.

*Trypsin treatment of purified mitochondria*: Sucrose gradient purified mitochondria were washed in Subcell Frac Buffer without PMSF and aliquoted into two samples. One sample was treated with 10 µg/ml trypsin and both samples were incubated for 30 min on ice with occasional gentle mixing. The trypsin was deactivated by the addition of 2 mM PMSF and the mitochondria were washed with Subcell Frac Buffer. Mitochondria were lysed as described in subcellular fractionation.

### Immunoblotting of yeast cell extracts

Equal amounts of yeast lysate fractions were resolved on 4–14% (4% stacking gel, 14% separating gel) Tris–Glycine SDS-PAGE gels and blotted onto a PVDF membrane (Bio-Rad) and immunostained with anti-SULT4A1 (12578-1-AP, Proteintech) first followed by stripping and staining with anti-Histone H3 (ab46765, Abcam), anti-GAPDH (GT239, GeneTex), or anti-VDAC1/porin (ab110326, Abcam) antibodies. Blots were visualized with Clarity Western ECL substrate (Bio-Rad) chemiluminescence and imaged using a gel-doc system (SYNGENE G:Box). All images were processed via Adobe Photoshop 2021 to correct signal levels of the complete image before cropping the shown area and placed into Adobe Illustrator 2021 to generate final figures. All samples shown in the figure panels were resolved in same gel.
